# Impact of mean monthly temperature on psychiatric admissions: data from an acute inpatient unit

**DOI:** 10.1192/j.eurpsy.2024.981

**Published:** 2024-08-27

**Authors:** N. Rizzo Pesci, S. Peracchia, E. Teobaldi, G. Maina, G. Rosso

**Affiliations:** ^1^Department of Neurosciences “Rita Levi Montalcini”, University of Turin, Turin; ^2^Unit of Psychiatry, San Luigi Gonzaga University Hospital, Orbassano (TO), Italy

## Abstract

**Introduction:**

Psychiatric disorders are large contributors to the global disease burden and their prevalence is increasing. Global climate is also facing changes, including a rise in temperatures. Many clinical conditions are affected by meteorological factors and there are numerous reports on the effect of climate changes on such conditions. Psychiatric disorders are also influenced by climatic factors but the literature on the effects of climate changes on mental health is limited.

**Objectives:**

The aim of this study is to investigate the impact of rising temperatures on the risk of acute exacerbation of psychiatric disorders.

**Methods:**

Data were collected retrospectively for a total of 139 months, *i.e.* from January 2012 to July 2023. Recordings of mean monthly temperatures were obtained from registries of the meteorological station of the Department of Physics of the University of Turin. For each of the 139 months, deviations from the average temperature of that month of any year were computed (ΔTm). Anonymised socio-demographic and clinical data on patients admitted during the observation period to the acute psychiatric unit of San Luigi Gonzaga University Hospital (Turin, Italy) were extracted from the hospital registry. Linear regression analyses were used for statistical analyses.

**Results:**

A total of 5420 admissions to our psychiatric ward were recorded over the observation period. Monthly deviations from average temperature and monthly number of admissions were directly correlated, with regression coefficient 1.803 (P = 0.0048) (Fig.1A). Linear regression analysis was performed between ΔTm and number of admissions according to diagnostic group. The regression coefficient was 0.1336 (P=0.5334) for admissions of patients with schizophrenia and related disorders (SCZ) (Fig.1B), 0.4575 (P=0.0295) for bipolar disorders (BD) (Fig.2A) and 0.3381 (P=0.0382) for major depressive disorder (MDD) (Fig.2B).

**Image:**

**Figure:**
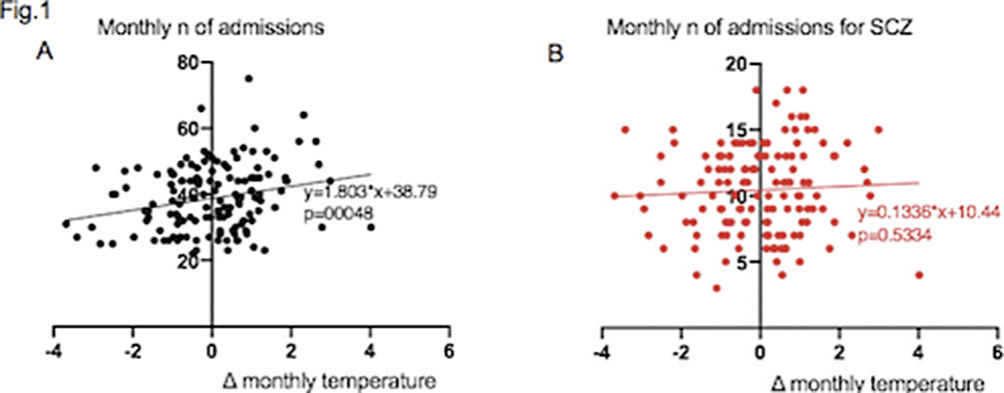

**Image 2:**

**Figure:**
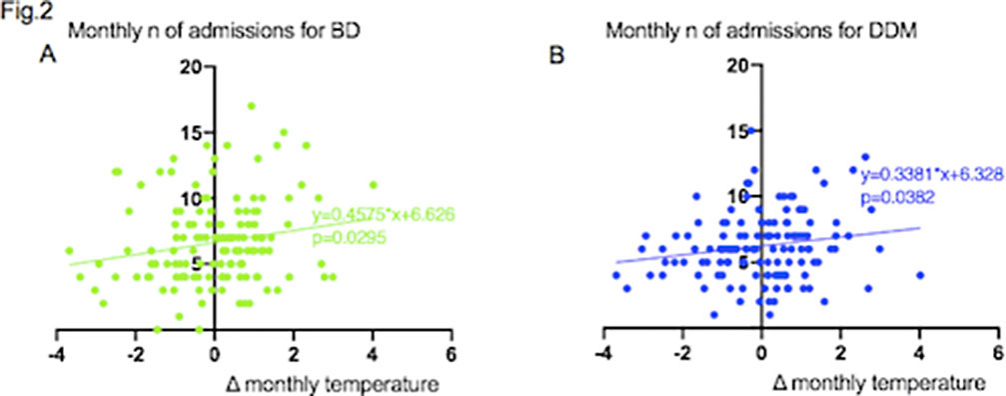

**Conclusions:**

These results confirm the impact of meteorological factors on mental disorders. In particular, we observed a positive correlation between monthly temperature and the number of admissions to our acute inpatient unit. The correlation was significant when taking into consideration admissions for exacerbation of bipolar disorder and major depressive disorder, but not when considering admissions for schizophrenia. This highlights the importance of climatic factors especially in mood disorders, provides new insights into their etiopathological mechanisms and provides information that can be implemented for follow up and relapse prevention.

**Disclosure of Interest:**

None Declared

